# Sexual reproductive healthcare utilisation and HIV testing in an integrated adolescent youth centre clinic in Cape Town, South Africa

**DOI:** 10.4102/sajhivmed.v19i1.826

**Published:** 2018-11-26

**Authors:** Andrea S. Mendelsohn, Katherine Gill, Rebecca Marcus, Dante Robbertze, Claudine van de Venter, Eve Mendel, Landisiwe Mzukwa, Linda-Gail Bekker

**Affiliations:** 1 The Desmond Tutu HIV Centre, University of Cape Town, South Africa; 2 Retreat Community Health Centre, Western Cape Department of Health, South Africa; 3 Desmond Tutu HIV Foundation Youth Centre, University of Cape Town, South Africa

## Abstract

**Background:**

HIV prevalence is increasing among South African youth, but HIV counselling and testing (HCT) remains low. Adolescent pregnancy rates are also high.

**Objectives:**

Innovative strategies are needed to increase HIV and pregnancy screening and prevention among youth.

**Method:**

The Desmond Tutu HIV Foundation Youth Centre (DTHF-YC) offers integrated, incentivised sexual and reproductive health (SRH), educational and recreational programmes. We compared HCT and contraception rates between the DTHF-YC and a public clinic (PC) in Cape Town to estimate the impact of DTHF-YC on youth contraception and HCT utilisation.

**Results:**

In 2015, females < 18 years had 3.74 times (confidence interval [CI]: 3.37–4.15) more contraception visits at DTHF-YC versus PC. There were no differences in the contraception and adherence was suboptimal. DTHF-YC youth (aged 15–24 years) were 1.85 times (CI: 1.69–2.01) more likely to undergo HCT versus PC, while male youth were 3.83 times (CI: 3.04–4.81) more likely to test at DTHF-YC. Youth were a third less likely to test HIV-positive at DTHF-YC versus PC. Female sex, older age, clinic attendance for contraception and sexually transmitted infections (STIs), redeeming incentives and high DTHF-YC attendance were all independent factors associated with increased HCT.

**Conclusion:**

Youth were significantly more likely to access SRH services at DTHF-YC compared with the PC. The differences were greatest in contraception use by female adolescents < 18 years and HCT by male youth. Increased HCT did not increase youth HIV case detection. Data from DTHF-YC suggest that youth-friendly healthcare providers integrated into community youth spaces may increase youth HCT and contraception rates.

## Introduction

South Africa has the highest prevalence of HIV infections among adolescents worldwide, accounting for nearly 18% of global HIV infections among 15- to 24-year-old youth in 2016.^1^ Although HIV incidence is decreasing, South Africa still had 9.9 new infections per 1000 adults in 2016, with approximately 37% of those new infections in young people aged 15–24 years (22% in young women).^1^ The HIV prevalence in a low socioeconomic, high-density township outside Cape Town was estimated to be 25% among residents ≥ 15 years of age in 2008 and 10.6% in 11- to 19-year-olds in 2006.^2,3^ With respect to other sexually transmitted infections (STIs), a female adolescent cohort (16–22 years; mean 18 years) in Cape Town found that > 70% tested positive for at least one STI or bacterial vaginosis; *Chlamydia trachomatis* prevalence alone was 42%.^4^ Survey data from four of nine South African provinces revealed that 19.2% of female adolescents (12–19 years) had been pregnant at least once^5^; similarly, the 2008 Youth Risk Behaviour Survey showed that 24% of female students (11–20 years) reported at least one pregnancy (majority unplanned).^6^

Despite the high prevalence of STIs and pregnancy, HIV testing, condom use and contraception coverage in South African youth remains suboptimal. A survey of 15- to 24-year-olds in KwaZulu-Natal found that only 29% of youth reported previous HIV counselling and testing (HCT),^7^ while a population-based survey conducted in four South African provinces documented that less than half of 18- to 24-year-old women used hormonal contraception.^8^ The same 2008 youth survey found that 30.7% of high school learners reported consistent condom use, while only 55% of students with STI symptoms had received treatment.^5^ Nationally, only 45.8% of the 15- to 24-year-old South African youth in 2016 were able to correctly identify ways of preventing sexual transmission of HIV.^1^

There remains a critical need to increase youth access to comprehensive SRH services in South Africa. Youth-friendly healthcare settings outside of traditional public clinics (PCs) may promote health-seeking behaviour and facilitate prevention and screening opportunities.

The Desmond Tutu HIV Foundation Youth Centre (DTHF-YC) has operated since 2011 in a resource-limited township in Cape Town, opposite the local secondary school, with extended after school hours. The Desmond Tutu HIV Foundation Youth Centre provides incentivised, adolescent-friendly health, educational and recreational activities for youth aged 12–23 years with the objective of widening access to HCT and SRH services.^9^ Youth at DTHF-YC can participate in formal educational or recreational programmes, access the computer laboratory, socialise in a safe space or use the SRH clinic (HCT, contraception, STI treatment, basic acute medical treatment). Youth are rewarded for healthy behaviours (e.g. HIV testing, STI treatment, contraception visits) with points (*tutus*) that are redeemable for rewards such as food or shopping vouchers at an exchange rate of three *tutus* per Rand (ZAR), but utilisation of SRH services is entirely voluntary.

To evaluate the impact of DTHF-YC on youth health-seeking behaviour, we compared HCT and contraceptive rates at DTHF-YC to those of youth at one of Cape Town’s PC in a township of similar demographics and healthcare access. In order not to stigmatise the communities, the two township names are kept anonymous.

## Methods

### Study setting and population

The Desmond Tutu HIV Foundation Youth Centre and PC both serve isolated low-income, high-density townships situated next to wealthier suburbs of Cape Town. Both townships began as informal settlements but have grown to include mixed formal and informal housing. In both communities, over 90% of people identify as Black African, 80% live on < R3200/month and only about 25% of the residents live in formal dwellings.^10,11^ Based on 2011 census data, both communities demonstrate a ‘youth bulge,’ with roughly 25% of the populations aged 10–24 years.^10,11^ PC population has grown since 2011 and was estimated at 40 000 – 60 000 by the police department based on aerial photographs. We used a conservative PC population estimate of 31327 residents based on city estimates of 5.5 people per service delivery point in informal settlements.^12^ The population serviced by the DTHF-YC was estimated at 21 904 residents in the city census, a number consistent with DTHF’s 2011 census.

With respect to healthcare access, both communities have a public clinic (PC) situated at the entrance of the township that provides basic SRH, HIV/TB and antenatal care services. The two public clinics had 1785 and 2354 HIV-infected people accessing treatment as of June 2016, which translates to 8.1% and 7.5% of the DTHF-YC and PC township’s respective populations.^13^ The residents of both communities have to travel 5 km to larger community health centres for general medical treatment. PC initiated a Friday afternoon clinic session facilitated by a family planning nurse to accommodate the youth after school. At other times, the youth can access adult SRH services. In the DTHF-YC township, the youth have access to a PC and DTHF-YC, which is located in a geographically separate facility opposite the secondary school and has extended afternoon hours Monday to Friday.

### Data source

Since 2011, DTHF-YC attendance, educational and recreational programme participation and *tutu* data have been collected via a biometric fingerprint tracking database in real-time on the visit day. Clinic visit data – including HCT testing and results, contraception type and pregnancy testing – are entered into the same DTHF-YC database by the attending nurse on the clinic visit day. For this cross-sectional study, we compared the total numbers of HIV tests and results, STI treatment and contraception visits at DTHF-YC from 01 January 2015 to 31 December 2015, with the equivalent services provided at PC (same age range and time period). The Desmond Tutu HIV Foundation Youth Centre data were extracted from the biometric data system for all youth who attended DTHF-YC in 2015. Clustered PC data were obtained from City of Cape Town Health Department records. Contraception data were compared for female adolescents < 18 years only, as the PC 18–23 year data were merged with all adult data in Cape Town records. HIV testing data were split according to gender and age categories of < 15 years and 15–24 years based on Cape Town records. PC HIV testing data included tests conducted as part of antenatal care in addition to routine screening tests. The Desmond Tutu HIV Foundation Youth Centre data included only non-pregnant youth who requested HIV testing. Male and female youth populations for the two townships were calculated by multiplying the total populations by the male:female ratios estimated as being within the age range of 10–24 years by the 2011 census.^10,11^

### Statistical analysis

In order to compare SRH utilisation at DTHF-YC versus the PC, HIV testing and contraception visit rates were calculated as the number of services provided (e.g. HIV tests and contraception visits) divided by the total number of youth estimated to be living in the two townships who could potentially access services. Two-way frequency tables and Chi-square tests were used to determine the effect DTHF-YC exposure had on HIV testing and contraception utilisation rates (95% confidence intervals [CIs]).

For DTHF-YC data only, each contraceptive visit was equated to 2–12 months of contraception coverage, depending on the method used. The total number of months of contraception coverage per woman per year was tabulated based on the number of visits and contraceptive type dispensed. Adherence was calculated as the number of months of coverage divided by 12 (perfect use was defined as continuous contraception through a single year).

For DTHF-YC data only, the association between HIV testing and DTHF-YC attendance with or without a clinic visit, incentives, contraception or STI treatment visits and demographic data was estimated using multivariable logistic regression. Age, DTHF-YC attendance and the number of incentive points redeemed for rewards were categorised as high or low based on the median. Quantitative data were analysed using STATA (Version 14, College Station, Texas, USA).

## Ethical considerations

Ethical approval was received from the University of Cape Town’s Faculty of Health Sciences Research Ethics Committee (HREC REF Number 015/2012) and the City of Cape Town (ID No: 10571).

## Results

In 2015, 2235 individual youth attended DTHF-YC. There were 22 430 total visits and 3143 DTHF-YC clinic visits (14.0%). The median age was 17.5 years (range 11.1–24.6, interquartile range [IQR] 15.2–19.8). Two thirds were female (1448 female [64.7%], 779 male [34.6%]). Individuals had a median of five visits/year to DTHF-YC (range 1–168, IQR 2–12). Three of these visits were spent socialising, with no formal activity (range 0–118 days; IQR 1–8). The remaining time was divided between formal educational or recreational programmes and clinic attendance. The DTHF-YC clinic provided 1084 HIV tests, 1932 contraception visits and treated 264 STIs in 2015. A median of 36 *tutus* were redeemed per person in exchange for food or vouchers (range 0–1200; IQR 0–202).

### Contraception utilisation

**FIGURE 1:**
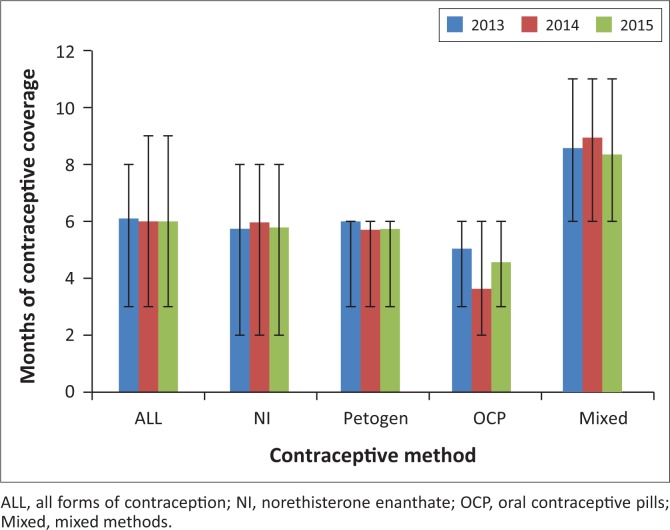
Desmond Tutu HIV Foundation Youth Centre contraception adherence in 12- to 24-year-old female youth. The median months per year of coverage for contraception among 15- to 24-year-old female youth are displayed by year and contraception type. Error bars indicate the interquartile range.

In 2015, the median age of female youth receiving contraception at DTHF-YC was 18.2 years (range 11.2–24.4, IQR 16.3–20.2). The Desmond Tutu HIV Foundation Youth Centre saw 712 individual females for contraception visits in 2015, accounting for 1932 visits. Female youth had a median of two contraception visits at DTHF-YC per year (IQR 1–4). In 2015, the median DTHF-YC female aged 15–24 years used contraception six months per year (IQR 3–9) (Figure 1, 50% contraception adherence). Median injectable contraceptive use was six months per year (norethisterone enanthate [Nur-Isterate] [IQR 2–8] and medroxyprogesterone acetate [Petogen] [IQR 3–6]); combined oral contraceptive coverage was less at three months per year (IQR 3–6). Women who used multiple contraceptive types in a year had a median of eight months of coverage per year (IQR 6–11). Age did not correlate with contraceptive adherence (*p* = 0.23). Contraceptive coverage per year did not change between 2013 and 2015 (mean use of six months/year for each year, Figure 1).

Adolescents under 18 years were 3.7 times (range: 3.37–4.15: *p* < 0.001) more likely to access contraception services at DTHF-YC versus PC (Table 1). Contraceptive use by type was consistent across sites. Most (84.2% and 97.3%) female adolescents favoured injectables at DTHF-YC and PC, respectively, with Nur-Isterate as the most common choice. Oral contraception and implants were uncommon at both sites.

**TABLE 1:** 2015 Contraception visits in female adolescents aged ≤ 18 years at Desmond Tutu HIV Foundation Youth Centre versus public clinic.

Contraception method	DTHF-YC *n* = 1577‡		PC *n* = 2036‡		Risk ratio	*p*
	Frequency (*n*)	Percentage (%)	Frequency (*n*)	Percentage (%)	(95% CI)	
Total contraception visits	1001	100.0	345	100.0	3.74 (3.37–4.15)	< 0.001
Oral contraceptive pills	22	2.2§	4	1.2	1.90 (0.66–5.46)	0.23
Norethisterone enanthate	764	76.3	285	82.6	0.92 (0.87–0.98)	0.015
Medroxyprogesterone acetate	79	7.9	51	14.7	0.34 (0.24–0.47)	< 0.001
Implant	11	1.1	5	1.4	0.76 (0.27–2.17)	0.61
Intra-uterine copper device	0	0	0	0	-	-
Emergency contraception	2	2.0	0	0	-	-
Unknown type†	123	12.3	0	0	-	-

DTHF-YC, Desmond Tutu HIV Foundation Youth Centre; PC, public clinic, CI, confidence interval.

† , Came for contraception, undocumented type administered;

‡ , *n* = 10- to 19-year-old females.

§ , % = per cent of contraception visits.

### Human immunodeficiency virus testing

The Desmond Tutu HIV Foundation Youth Centre performed 1084 HIV tests for youth in 2015: (671/2235 [30.0%] youth underwent at least one test; Table 2). The median age of those tested was 18.0 years (range 11.2–23.6, IQR 15.7–20.0); each individual had a median of one HIV test/year (IQR 1–2). Youth at DTHF-YC were 1.85 times more likely to get an HIV test versus PC (CI: 1.69–2.01, *p* < 0.001) (Table 2). Female adolescents < 15 years and male youth aged 15–24 years were 2.23 times more likely (CI: 1.68–3.04, *p* < 0.001) and 3.83 times more likely (CI: 3.04–4.81, *p* < 0.001) to test at the DTHF-YC versus PC, respectively.

**TABLE 2:** 2015 Desmond Tutu HIV Foundation Youth Centre versus public clinic HIV testing.

Gender	Age (years)	DTHF-YC		PC		Comparison	
		HCT visits	No HCT	HCT visits	No HCT	Risk ratio (95% CI)	*p*
Male	10–14	48	565	47	861	1.51 (1.03–2.23)	0.04
	15–24	274	2114	93	3009	3.83 (3.04–4.81)	< 0.001
Female	10–14	101	556	64	876	2.23 (1.68–3.04)	< 0.001
	15–24	661	1880	556	2514	1.44 (1.30–1.59)	< 0.001
**Total**	**10–24**	**1084**	**5115**	**760**	**7260**	**1.85 (1.69–2.01)**	**< 0.001**

DTHF-YC, Desmond Tutu HIV Foundation Youth Centre; PC, public clinic; HCT, HIV counselling and testing; CI, confidence interval.

More youth tested HIV-positive at PC than at DTHF-YC (41 [5.4%] versus 19 [2.1%] in 2015, respectively Table 3). PC youth were 3.13 times more likely to have a positive HIV test versus DTHF-YC (CI: 1.67–5.26, *p* < 0.001). In both clinics, the majority of HIV-positive tests were in female youth aged 15–24 years (16/19 [84%] and 39/41 [95%] of DTHF-YC and PC HIV-positive tests, respectively).

**TABLE 3:** 2015 Desmond Tutu HIV Foundation Youth Centre versus public clinic HIV Counselling and testing results.

Gender	Age (years)	DTHF-YC				PC				Comparison	
		HIV-positive		HIV-negative		HIV-positive		HIV-negative		Risk ratio	*p*
		Frequency (*n*)	Percentage (%)	Frequency (*n*)	Percentage (%)	Frequency (*n*)	Percentage (%)	Frequency (*n*)	Percentage (%)	(95% CI)	
Male	10–14	0	0.0	48	100.0	0	0.0	47	100.0	-	-
	15–24	2	0.1	272	99.3	2	2.2	91	97.8	0.34 (0.05–2.38)	0.25
Female	10–14	1	0.0	100	99.0	0	0.0	64	100.0	-	-
	15–24	16	2.4	645	97.6	39	7.0	517	93.0	0.35 (0.19–0.61)	< 0.001
**Total**	**10–24**	**19**	**2.1**	**1046**	**98.2**	**41**	**5.4**	**719**	**94.6**	**0.32 (0.19–0.56)**	**< 0.001**

DTHF-YC, Desmond Tutu HIV Foundation Youth Centre; PC, public clinic.

### Significant predictors of Human immunodeficiency virus testing

The most significant predictor of HIV testing at DTHF-YC was obtaining STI treatment (Table 4). Symptomatic youth who received STI treatment were 2.69 times more likely to have an HIV test (CI: 1.92–3.79, *p* < 0.001) versus no STI management. Similarly, female youth seen at DTHF-YC for contraception were 1.97 times more likely to undergo HIV testing versus those who did not have a contraception visit (CI: 1.54–2.52, *p* < 0.001). Female youth were 1.58 times more likely to test than males (CI: 1.22–2.04, *p* < 0.001), while youth > 17 years of age were 1.43 times more likely to test than youth < 17 years (CI: 1.16–1.76, *p* < 0.001). Frequent DTHF-YC visitors (> 5 visits) were 2.17 more likely to test than infrequent visitors (CI: 1.69–2.77, *p* < 0.001). Similarly, those who redeemed a greater number of incentive points for food or vouchers were 1.75 times more likely to test than those with below median incentive use (CI: 1.38–2.21, *p* < 0.001).

**TABLE 4:** Multivariable logistic regression analysis on the effect of independent variables on HIV counselling and testing (exposed to HIV counselling and testing) versus not HIV testing (unexposed to HIV counselling and testing) at the Desmond Tutu HIV Foundation Youth Centre in 2015.

Variable	HCT performed^a^		HCT not performed		Total	Adjusted OR (CI)	*p*
	Frequency (*n*)	Percentage (%)	Frequency (*n*)	Percentage (%)			
**Sex**							
Female	517	35.6	936	64.4	1453	1.58 (1.22–2.04)	< 0.001
Male	154	19.7	628	80.3	782	-	-
**Age (years)**							
12–16	252	25.6	732	74.4	984	-	-
17–23	419	33.5	832	66.5	1251	1.43 (1.16–1.76)	< 0.001
**Attendance**							
Low (< 5 visits)	203	19.0	866	81.0	1069	-	-
High (≥ 5 visits)	468	40.1	698	59.9	1166	2.17 (1.69–2.77)	< 0.001
**Incentive use**							
Low (< 35 tutus redeemed)	212	19.0	902	81.0	1114	-	-
High (≥ 35 tutus redeemed)	459	40.9	662	59.1	1121	1.75 (1.38–2.21)	< 0.001
**Contraception visit^b^**							
Yes	345	48.5	367	51.5	712	1.97 (1.54–2.52)	< 0.001
No	326	21.4	1197	78.6	1523	-	-
**STI visit**							
Yes	98	55.1	80	44.9	178	2.69 (1.92–3.79)	< 0.001
No	573	27.9	1484	72.1	2057	-	-
**Total YC participants**	**671**	**30.0**	**1564**	**70.0**	**2235**	-	-

HCT, HIV counselling and testing; YC, youth centre; STI, sexually transmitted infection; OR, odds ratio; CI, confidence interval.

† , HCT performed, had at least 1 HIV test in 2015; HCT not performed, no HIV test in 2015.

‡ , Females only.

## Discussion

The DTHF-YC created an integrated health, educational and recreational programme in order to increase youth access to comprehensive SRH services. The PC had made a number of adolescent-friendly adaptations to increase youth utilisation of health services given the staffing, work hours and budget constraints of a public clinic. We compared the two clinics to test our hypothesis that exposure to an incentivised, integrated youth centre and clinic would increase youth utilisation of SRH services, with a primary focus on increasing access to HIV testing and contraception.

We demonstrated that youth adolescent healthcare utilisation was markedly higher at DTHF-YC in comparison with PC. Nearly four times more female adolescents under 18 years had contraception visits at DTHF-YC versus PC. Although DTHF-YC had more contraception visits, patients at both clinics opted for similar types of contraception (injectables). Similarly, implant and intra-uterine device use was low in both clinics. Intensive community outreach may be needed to increase youth interest in the more effective implant and intra-uterine contraception options.

Despite increased use of contraception at DTHF-YC, adherence was poor (average female yearly use at DTHF-YC was approximately 50%). Reasons given during informal discussions included forgotten appointments, too busy to return to clinic (despite ‘adolescent-friendly’ hours), travel outside the province or interruption of contraception between relationships. This contraception adherence pattern has implications for how pre-exposure prophylaxis (PrEP) might be used by an adolescent female population if it were readily available in the South African public sector alongside contraception as a part of a HIV prevention package. Given that PrEP requires up to seven days of daily dosing to reach adequate levels, it may be ineffective for adolescent females to cycle on and off PrEP as they do for contraception as most female patients only restarted contraception after a new relationship commenced.^14,15^ Public health education strategies should engage adolescent girls about the benefits of using both contraception and PrEP continuously until they have a more prolonged period of either abstinence or monogamy to maximise prevention strategies.

The DTHF-YC model successfully increased the rate of youth HIV testing, particularly in male youth. Nearly twice the numbers of HIV tests were performed at DTHF-YC than PC, with nearly four times the number of tests in males. The Desmond Tutu HIV Foundation Youth Centre introduced the following youth-friendly services: (1) extended hours five days per week, (2) dedicated youth-friendly nurses with decreased wait times, (3) geographic separation from adult services, (4) close proximity to the high school, (5) a safe and fun space for youth to spend time, (6) free computer access, (7) structured extracurricular activities and (8) an incentive programme linked to testing and contraception. We believe that these combined factors contribute to the DTHF-YC’s successful increase in HIV testing and contraception utilisation. We believe that the number of HIV tests for female youth at PC was falsely elevated, as that number included 2–3 tests provided as part of routine antenatal care. We believe that the difference in HIV testing for female youth between DTHF-YC and PC would have been greater had the data included only voluntary testing outside of antenatal care. Nonetheless, the increased HIV testing in male youth demonstrated that DTHF-YC successfully increased HIV testing and healthcare utilisation in young males, a notoriously difficult to reach population. Most male youth only go to PCs if they are ill, whereas at DTHF-YC healthy males came to socialise or participate in a programme. Once they were at DTHF-YC, they were more likely to test.

There may have been multiple factors associated with increased HIV testing at DTHF-YC, suggesting that no single strategy can be deployed to increase HCT in youth. Older youth were more likely to test (consistent with increased sexual activity and increased HIV risk). Female youth were also more likely to test than male youth, perhaps owing to increased clinic attendance for contraception. Patients attending for contraception and STI treatment were more likely to get an HIV test, supporting the notion that youth want a comprehensive package of SRH services. High attendance and incentive use were also associated with increased testing, but many youth who redeemed incentive points still chose not to test. Incentives alone are likely insufficient to promote HIV testing. A less costly variation of DTHF-YC’s model may be created by placing publicly funded SRH youth services in community spaces in which youth currently ‘hang out,’ such as schools, libraries and sports facilities, during convenient after school and weekend hours. However, for non-clinic-based SRH services to be successful like the DTHF-YC they need to be consistent, private and permanent in order to gain youth trust over time; we do not believe that periodic community campaigns will have the same effect.

Although DTHF-YC increased youth testing, it was not a perfect strategy. Despite the youth-friendly services, only 30% of the youth that came to DTHF-YC in 2015 received an HIV test. Not all of these youth were sexually active or at risk. However, given the median age of sexual debut for girls and boys in South Africa as 16 and 15 years, respectively,^16^ we suspect that > 30% of youth at DTHF-YC are at risk for HIV. Alarmingly, 45% of youth who presented for STI treatment declined HIV testing, suggesting that more stigma or fear remain associated with HIV than other STIs. Further investigation is warranted to understand this lack of testing and the impact of community stigma or risk denial on a youth’s decision to test.

Finally, HIV testing as a prevention strategy assumes that testing will lead to increased case detection and HIV treatment. Interestingly, even though DTHF-YC did more HIV tests, PC diagnosed three times the number of new HIV cases. Given that the two communities have comparable demographics and population subsets retained in HIV care, we would expect similar HIV prevalence in the two populations. PCs with active HIV treatment programmes and are likely testing sicker youth who may present with other opportunistic infections, whereas DTHF-YC does more routine testing of healthy individuals. There is certainly value in HCT irrespective of the result. Habituating HIV testing in a younger population may make routine testing more likely as that population ages, particularly in hard-to-reach male youth. However, our data suggest that increased non-targeted testing of healthy youth is not a high yield strategy for finding undiagnosed HIV cases among the youth.

In addition, we suspect that DTHF-YC attracts an in-school youth population who may be less at risk than their out-of-school peers. HIV testing at PC for healthy women is often linked to mandatory antenatal care, supporting the theory that healthy non-pregnant youth who choose to test might be at lower risk than the general population. All South African youth need access to friendly healthcare services. The Desmond Tutu HIV Foundation Youth Centre successfully increased HIV testing and contraception utilisation for sexually active youth who sought healthcare, irrespective of HIV risk. Nonetheless, additional community outreach and non-clinic-based strategies should be employed to reach the most vulnerable out-of-school or unemployed youth who may not attend youth centres or clinics, no matter how convenient.

## Limitations of the study

This study has several limitations. Because PC data were only available at the unit of services provided (HIV test, family planning visit), a comparison at the individual level was not possible. As a result, the width of our confidence intervals may be underestimated owing to our inability to account for clustering. Nonetheless, since tested individuals at DTHF-YC had a median of one HIV test per year, we suspect that clustering would have had minimal impact on the HIV testing data. There was more than one contraception visit per person per year. However, given the high degree of statistical significance of our findings, it seems unlikely that clustering would have qualitatively changed our contraception results. Finally, as HIV testing is not rare, the reported odds ratios might actually present a less accurate approximation of the risk ratio of the tested independent variables.

## Conclusion

In conclusion, our data suggest that convenient, confidential, youth-friendly SRH services associated with youth social spaces and activities can increase healthcare utilisation, specifically contraception and HCT. Innovative strategies such as community or school-based outreach programmes that include contraception, HCT and SRH services, including PrEP, are needed to blend the success of the DTHF-YC with existing public facilities and healthcare workers.
